# Assessment of pre and postinfection immunity to the hemagglutinin proteins of Influenza A(H1N1)pdm09 and Influenza A(H3N2) in India: a retrospective longitudinal study

**DOI:** 10.1186/s12985-026-03146-w

**Published:** 2026-04-05

**Authors:** Anup Jayaram, Desmond Cardoso, Arun M.T., Prasad Varamballi, Kavitha Karunakaran, Prachi Malsane, Sudheesh N., Ujwal Shetty, Naren Babu N., Chiranjay Mukhopadhyay, Anitha Jagadesh

**Affiliations:** Manipal Institute of Virology, Manipal Academy of Higher Education, Manipal, 576104 Karnataka India

**Keywords:** Influenza virus, Humoral immune response, Natural infection, HA-specific antibodies, Preexisting antibodies, Cross-reactive antibodies, Good Health

## Abstract

**Background:**

Hemagglutinin (HA), one of the main surface glycoproteins of the influenza virus, is essential for viral entry into host cells. Antibodies targeting HA can neutralize the virus, offering protection against infection. HA-specific antibody titers are therefore key markers of immune protection and vaccine efficacy. However, data on humoral immune responses to influenza A(H3N2) and influenza A(H1N1)pdm09 and viruses in India remain limited.

**Methodology:**

Laboratory-confirmed influenza specimens from 2016 to 2017 were analyzed in this retrospective longitudinal study. A systematic random sampling method was used to obtain 160 paired serum samples, distributed among four distinct age groups. Hemagglutination inhibition (HAI) assays were performed to assess the antibody responses against influenza A(H3N2) and influenza A(H1N1)pdm09 viruses following natural infection. Additionally, preexisting antibodies and antibodies to heterologous influenza strains were evaluated.

**Results:**

Following natural infection, seroconversion rates were 77.1% for individuals infected with influenza A(H3N2) and 84.9% for those infected with influenza A(H1N1). Preexisting antibodies were detected in 40.8% of the acute-phase influenza A(H3N2) samples and 17% of the acute-phase influenza A(H1N1)pdm09 samples. Antibodies to heterologous strains were also detected in both groups, particularly between influenza A(H1N1)pdm09 and influenza A(H3N2), as well as with influenza B lineages.

**Conclusion:**

The results of this study show that most of the study population had evidence of immunity against both influenza A(H3N2) and influenza A(H1N1) virus strains. Evidence of antibodies to other influenza strains was also observed in a few samples, thus emphasizing the need for continuous monitoring and tailored vaccination strategies in India.

**Supplementary Information:**

The online version contains supplementary material available at 10.1186/s12985-026-03146-w.

## Introduction

Influenza virus is an enveloped RNA virus that belongs to the *Orthomyxoviridae* family [1]. It causes a global burden of approximately one billion cases and 290,000–650,000 deaths annually [2]. The virus spreads through direct contact with an infected individual and through respiratory droplets. It can cause respiratory infections that can lead to severe illness, particularly in young children, older people, and those with underlying health conditions [2–4]. Influenza A and B are the major subtypes that infect humans and cause seasonal outbreaks [5].

Hemagglutinin (HA) and neuraminidase (NA) are the primary surface glycoproteins responsible for virus entry and exit. Influenza viruses evade host immunity through antigenic drift, which leads to alterations in HA and NA proteins and the emergence of novel strains [6, 7]. Currently, 18 hemagglutinin (HA) and 11 neuraminidase (NA) subtypes are recognized among influenza A viruses, with influenza A(H1N1) and influenza A(H3N2) being the predominant strains that infect humans, influencing seasonal flu patterns. Vaccination continues to be the most effective and essential strategy for preventing influenza virus infection [4]. However, because of antigenic drift, the influenza vaccine formulation needs to be revised annually on the basis of World Health Organization (WHO) surveillance data to ensure effective protection [8].

Upon influenza virus infection, the humoral immune system develops antibodies that respond to different viral surface glycoproteins. Among these, HA-specific antibodies help prevent illness by neutralizing the virus [9]. These antibodies bind to the head domain of HA, which contains the receptor-binding site necessary for viral attachment to sialic acid receptors on host cells. HA-specific antibodies prevent the virus from initiating infection by blocking this interaction through steric hindrance or mimicking the sialic acid structure [6, 10, 11]. Hemagglutination inhibition (HAI) assays can be used to measure this neutralizing activity.

Protective immune responses are generated by neutralizing antibodies targeting causative strains as well as strains with similar antigenic properties in response to natural influenza virus infection [12]. Most studies examining the immune response to influenza focus on the antibody profile postvaccination. However, the immune response following natural infection, particularly in diverse populations, is less understood. In India, influenza surveillance is focused primarily on influenza A(H1N1), as it is a notifiable disease under the Integrated Disease Surveillance Programme (IDSP) administered by the Ministry of Health and Family Welfare, Government of India (MoHFW). The burdens of influenza A(H3N2) and influenza B are unknown because of a lack of data [13]. While some surveillance data exist, very little information is available regarding the breadth of the humoral immune response following natural influenza infection in the Indian population. Therefore, we aimed to assess the humoral immune response targeting influenza A(H3N2) and influenza A(H1N1)pdm09 after natural infection across different age groups and to determine preexisting immunity and antibodies to other influenza A/B strains using the hemagglutination inhibition (HAI) assay.

## Methodology

### Study design and population

A retrospective longitudinal analysis was performed on serum samples obtained from the “Hospital-based Surveillance of Acute Febrile Illness (AFI)” study in India conducted at the Manipal Institute of Virology (MIV) [14]. The AFI surveillance project was conducted in 10 states across India, but for this study, we included four states, Karnataka, Kerala, Tamil Nadu, and Assam, where representative samples were available across all age groups. Nasopharyngeal and throat swabs were collected from patients with influenza-like illness (ILI), and RNA extracted from these samples was tested for influenza A(H3N2) and influenza A(H1N1)pdm09 by real-time reverse transcription polymerase chain reaction (RT‒PCR) using a standardized World Health Organization (WHO) protocol. Only laboratory-confirmed samples for influenza A(H3N2) and influenza A(H1N1)pdm09 were included in the study. Serum samples collected between 2016 and 2017 were used for serological testing. The acute-phase samples were collected within 1–5 days after symptom onset, and the follow-up samples were collected 4–6 weeks after infection from the same individuals. The study population was stratified into four age groups: 5–15 years, 16–30 years, 31–50 years, and 51–65 years. A total of 10 acute and 10 follow-up (4–6 weeks post-infection) samples were selected per age group from four states, yielding 40 samples per state. A systematic random sampling method was used for the selection of samples. A total of 160 samples were collected across the four states for each influenza A subtype (A(H3N2) and A(H1N1)pdm09). Age-matched influenza A/B-negative sera were included as assay controls for each age group to determine the background reactivity.

**Fig. 1:**
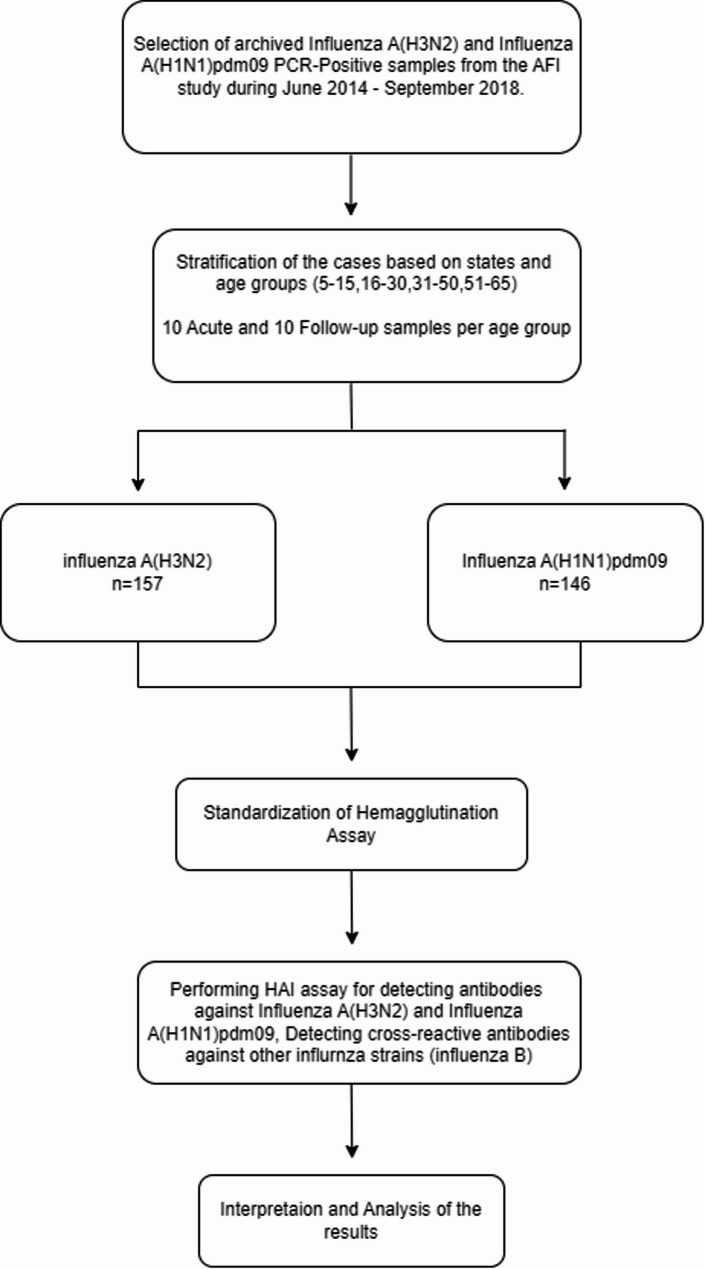
Flowchart of methodology

Among 160 samples, 157 influenza A(H3N2) and 146 influenza A(H1N1)pdm09 positive samples were included for the analysis due to limited sample volumes. The detailed workflow and sampling method are illustrated in Fig. 1.

.

### Viruses and virus propagation

The influenza vaccine strains (Northern Hemisphere) recommended by the WHO for the 2017–2018 season were used to evaluate the antibody response for the samples collected during the year 2016–2017, ensuring antigenic relevance to strains prevalent at that time. This approach aligns with global surveillance practices, where vaccine formulations for the upcoming influenza season are based on strains that circulated during the previous year. Table 1 summarizes the details of the influenza viruses used for this study.

**Table 1:** Influenza virus strains used for the HAI assay

Year	Subtype	Circulating strains
**2016–2017**	**Influenza A(H1N1)**	A/Michigan/45/2015 (H1N1) pdm09-like virus
	**Influenza A(H3N2)**	A/Hong Kong/4801/2014 (H3N2)-like-virus A/Singapore/INFIMH-16–0019/2016 (H3N2)-like virus
	**Influenza B (Victoria)**	B/Brisbane/60/2008-like virus B/Colorado/6/2017-like virus
	**Influenza B (Yamagata)**	B/Phuket/3073/2013-like virus

The Madin–Darby canine kidney (MDCK) cell line was used to propagate the influenza viruses. The viruses were grown under standard conditions, harvested, and stored at −80 °C, following World Health Organization (WHO) protocols for virus propagation [15].

### Hemagglutination (HA) and hemagglutination inhibition (HAI) assays

Hemagglutination assays were performed according to WHO protocols for influenza A(H3N2), influenza A(H1N1)pdm09, and influenza B (Yamagata and Victoria) viruses [16]. In a 96-well U-bottomed plate (Corning^®^), twofold dilutions of the virus were prepared using phosphate-buffered saline (PBS). After the dilution, 25 µL of a 1% human O-positive red blood cell (RBC) cell suspension was added to all wells, mixed gently, and kept for 45 min at room temperature. The HA titer was recorded as the highest dilution of virus showing complete hemagglutination. The hemagglutination titer was standardized to 4 HA units for the hemagglutination inhibition (HAI) assay.

Before performing HAI assay, serum samples were first treated with receptor-destroying enzyme (RDE) for 16–18 h at 37 °C to remove nonspecific inhibitors, followed by heat inactivation at 56 °C for 30 min. Twofold dilutions of the serum samples were prepared in U-bottom 96-well plates (Corning^®^), starting at a dilution of 1:10 and continuing up to 1:20,480. Afterward, 25 µL of 4 HA units was added, and the plates were incubated at room temperature (for 15 min). Subsequently, 50 µL of a 1% human O+ blood RBC cell suspension was added, and the plates were incubated for an additional 45 min. The HAI titer was defined as the highest serum dilution that completely inhibited hemagglutination.

Serum negative for influenza A/B were used as negative controls for each age group. We quantified antibody titers following adjustment for background, and the geometric mean titer (GMT) was evaluated for acute-phase and follow-up samples. A HAI titer of ≥ 40 was considered indicative of a significant immune response, which is consistent with the WHO criteria for seroprotection [17, 18]. Samples with acute-phase titers ≥ 40 were categorized as having preexisting antibodies. Serum samples with a ≥ 4-fold increase in the HAI antibody titer between the paired acute-phase and follow-up phase serum samples were classified as seroconverted [19, 20].

### Statistical methods

GraphPad Prism 8.0.0 (San Diego, CA) was used for data analysis. To assess the associations between clinical symptoms and age groups in both influenza A(H3N2) and influenza A(H1N1)pdm09-positive patients, a chi-square (χ²) test was conducted. The Wilcoxon signed-rank test was employed to compare HAI titers across paired acute and convalescent serum samples. The threshold for statistical significance was set at *p* < 0.05. Additionally, a 2-way ANOVA with multiple comparisons was performed to examine the significance across different age groups.

## Results

### Clinical characteristics of the study participants

**Fig. 2:**
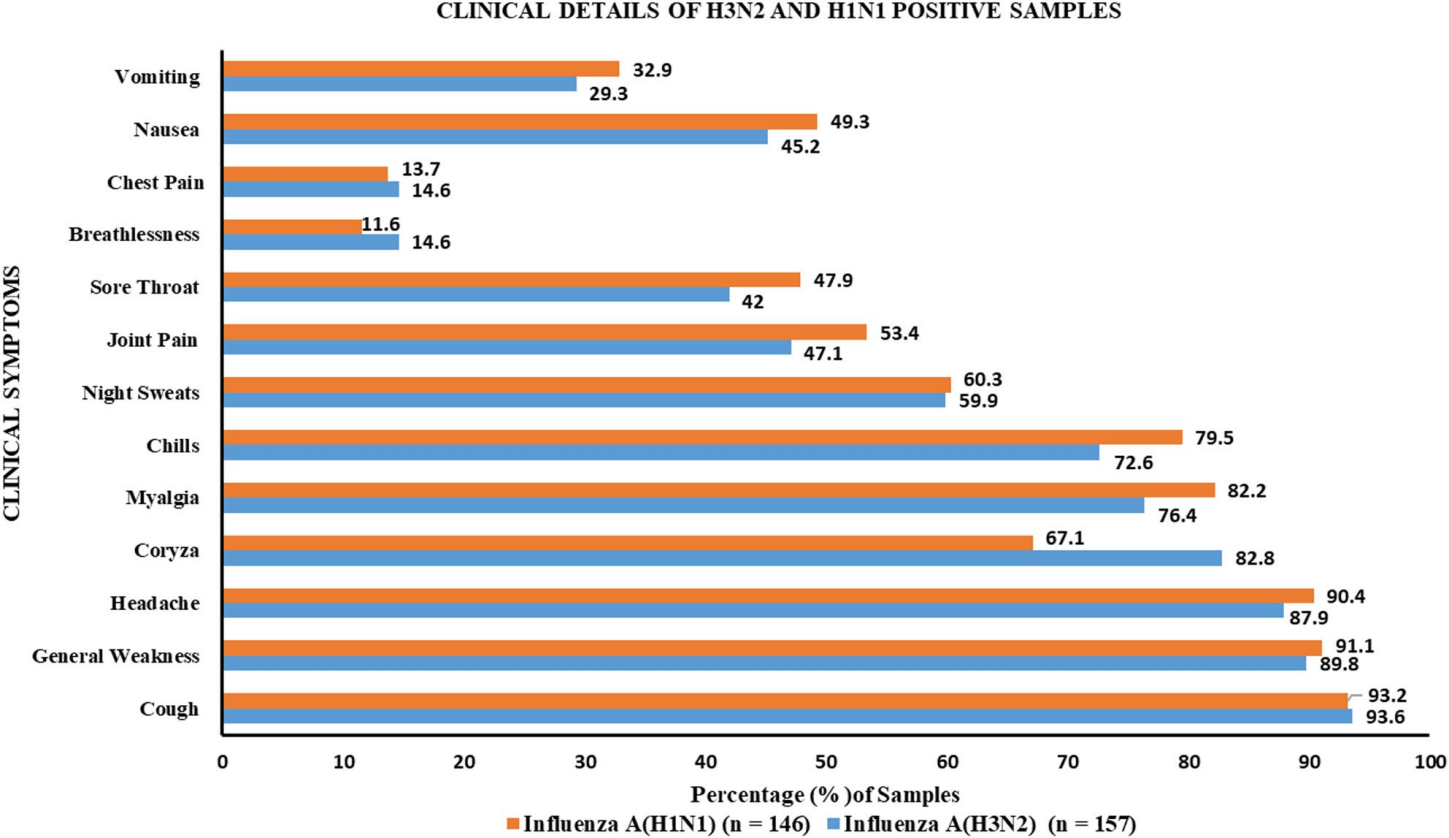
Comparison of clinical symptoms between influenza A(H3N2)- and influenza A(H1N1)-positive samples. The Y-axis represents clinical symptoms, whereas the X-axis indicates the percentage of samples exhibiting each symptom. Influenza A(H1N1) is represented by an orange bar, and influenza A(H3N2) is represented by a blue bar. Sample sizes: influenza A(H1N1) (*n* = 146) and influenza A(H3N2) (*n* = 157)

The clinical characteristics of the study population are summarized in Fig. 2. The most reported symptoms for both groups were cough (influenza A(H3N2): 93.6%, influenza A(H1N1): 93.2%), headache (influenza A(H3N2): 87.9%, influenza A(H1N1): 90.4%), and general weakness (influenza A(H3N2): 89.8%, influenza A(H1N1): 90.4%). Coryza was more common in influenza A(H3N2)-positive patients (82.8%) than in influenza A(H1N1)-positive patients (67.1%), whereas myalgia and chills were slightly more prevalent among influenza A(H1N1) patients (82.2% and 79.5%) than among influenza A(H3N2) patients (76.4% and 72.6%).

The clinical symptoms of influenza A(H1N1) and influenza A(H3N2) were analyzed across four age groups (5–15, 16–30, 31–50, and 51–65 years). Symptoms such as chills, sore throat, cough, chills, and general weakness were consistently prevalent across all age groups and subtypes. Among upper respiratory symptoms, coryza was found to be more common in influenza A(H3N2)-positive patients (80–85%) than in influenza A(H1N1)-positive patients (60–75%). Older individuals (51–65 years) experienced breathlessness, chest pain, and joint pain, indicating potentially more severe disease manifestations in this group. Clinical symptoms were analyzed among different age groups, and no significant difference was observed between influenza A(H1N1) and influenza A(H3N2) patients (*Supplementary Table 1*).

### Hemagglutination inhibition (HAI) response

#### HAI response to influenza A(H3N2) in acute and follow-up samples

The HAI antibody response to influenza A(H3N2) virus was evaluated in 157 paired acute and follow-up serum samples. In the acute phase, 64 samples (40.8%) presented HAI titers of ≥ 40, whereas 121 samples (77.1%) demonstrated HAI titers of ≥ 40 in the follow-up phase. This substantial increase in HAI titers between the two phases was confirmed to be statistically significant by the Wilcoxon signed-rank test, reflecting the development of a robust serological response following natural infection (*Supplementary Fig. 1A*).

**Fig. 3:**
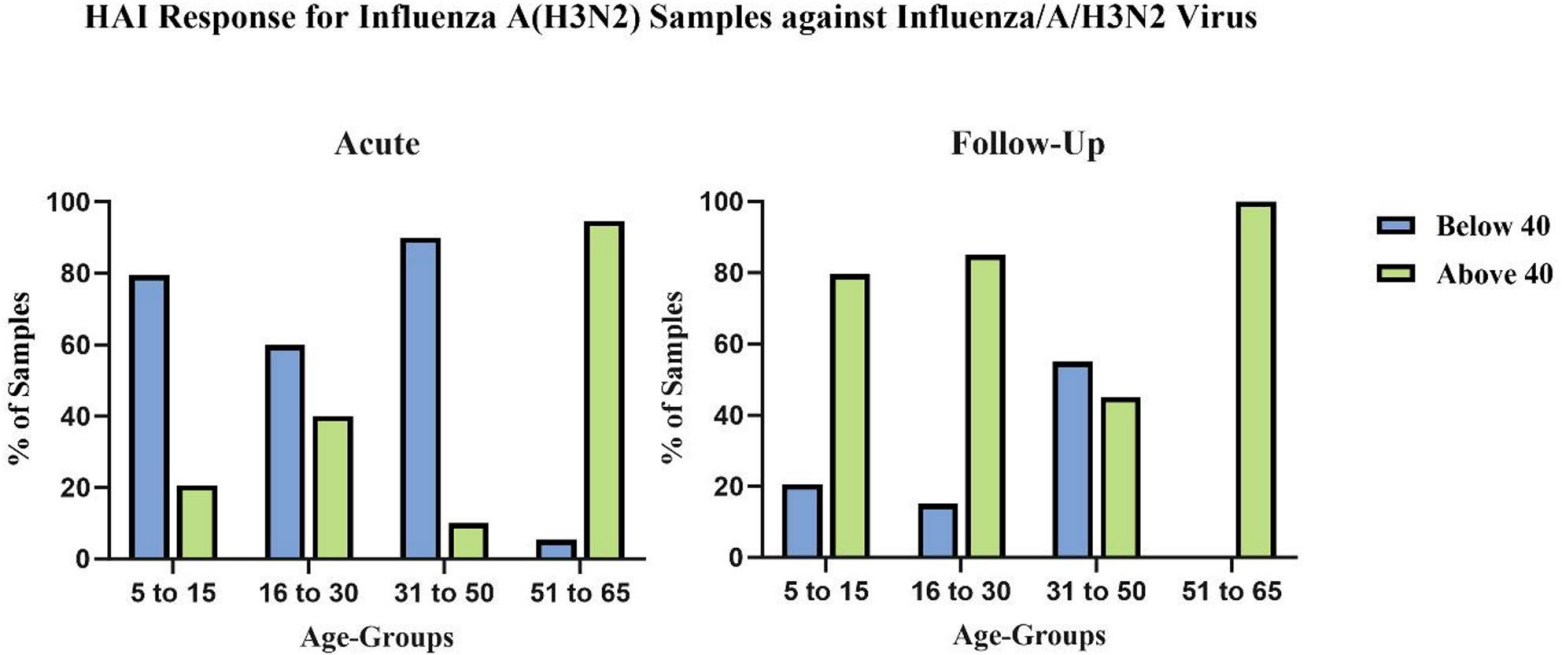
Age-stratified HAI Titer among Influenza A(H3N2)-positive samples: (**A**) Acute Samples and (**B**) Follow-up Samples. The X-axis shows age groups; the Y-axis shows the percentage of samples with HAI titers More than or equal to 40 (≥ 40) and below 40 (< 40)

Age-specific analysis revealed notable differences in the HAI titer for each age category in the acute and follow-up phases (Fig. 3). In the acute phase, 20.5% (8/39) of the samples from the 5–15 year age group, 40% (16/40) of those from the 16–30 year age group, 10% (4/40) of those from the 31–50 year age group, and 94.7% (36/38) of those from the 51–65 year age group presented HAI titers ≥ 40. In the follow-up phase, the corresponding titers increased to 79.5% (31/39), 85% (34/40), 45% (18/40), and 100% (38/38), respectively.

**Fig. 4:**
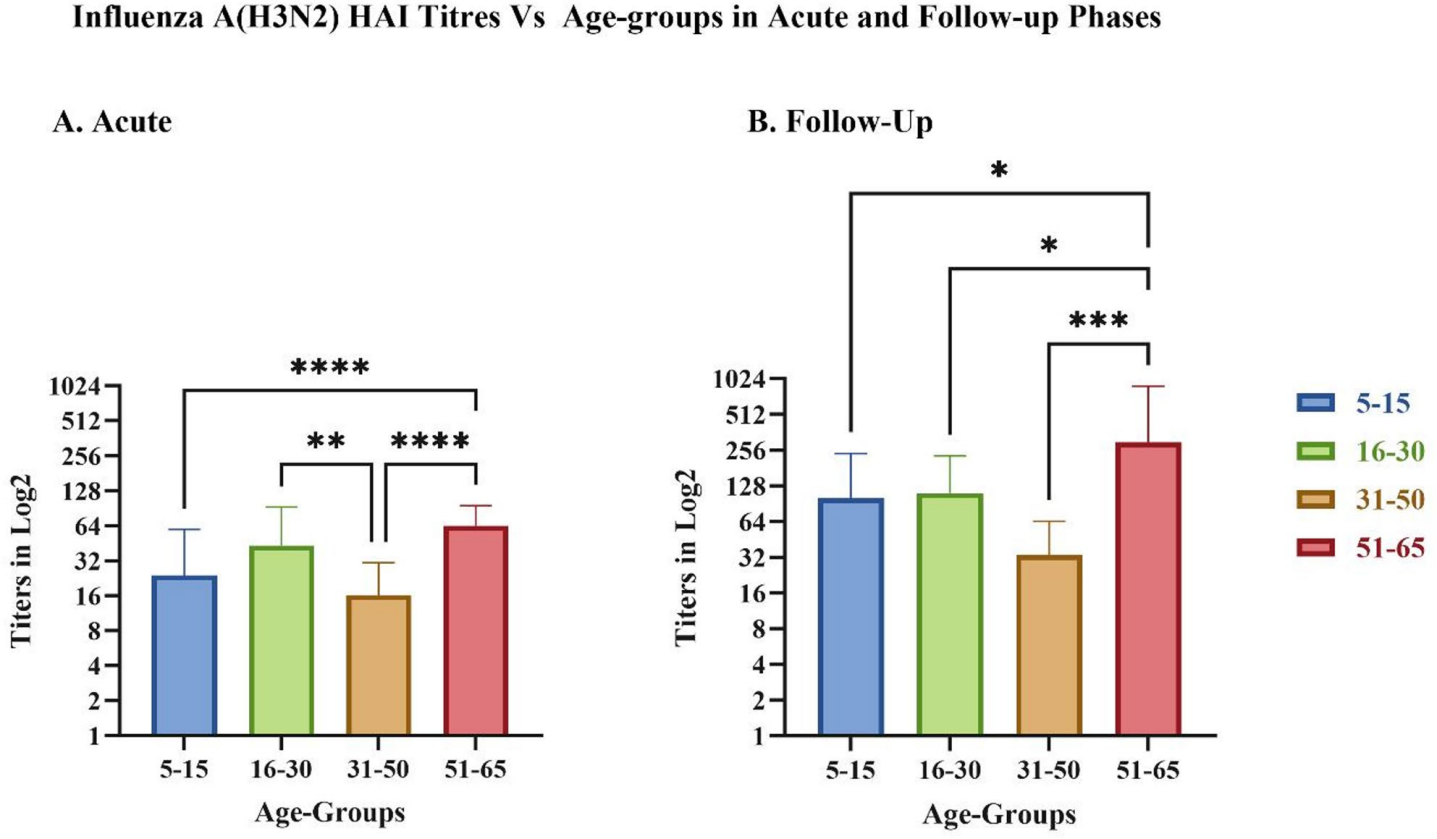
Hemagglutination inhibition (HAI) titers across age groups in influenza A(H3N2)-positive samples. Bar graphs display HAI titers (log₂ scale) stratified by different age groups in (**A**) acute-phase and (**B**) follow-up samples. Statistical significance between age groups was assessed using two-way ANOVA with Tukey’s multiple comparisons denoted by asterisks (*p < 0.05, **p < 0.01, ***p < 0.001, ****p < 0.0001)

A detailed age-stratified analysis of HAI titers is shown in Fig. 4. In the acute phase (Fig. 4A), the HAI titers were significantly higher in the oldest age group (51–65 years) than in the other age groups. In the follow-up phase (Fig. 4B), titers increased across all age groups, with older age groups showing higher titers. Some follow-up samples had titers of up to 1:1280, indicating a strong immune response (*Supplementary Fig. 2*). Statistical analysis using two-way ANOVA and Tukey’s multiple comparison test confirmed significant differences in titer levels between both age groups and sampling time points (acute vs. follow-up). (Note: Two H3N2 strains were used for HAI, but owing to minimal differences in antibody responses, the data were combined and are presented together in Figs. 3 and 4, and Supplementary Fig. 2.)

#### HAI response to influenza A(H1N1) in acute and follow-up samples

A total of 146 paired acute and follow-up serum samples positive for influenza A(H1N1) were analyzed using a hemagglutination inhibition (HAI) assay. Among these samples, only 25 (17.1%) acute samples and 124 (84.9%) follow-up samples presented HAI titers of ≥ 40. A Wilcoxon test confirmed that this increase was statistically significant *(Supplementary Fig. 1B).* Unlike individuals infected with influenza A(H3N2), whose baseline titers were relatively high, individuals infected with influenza A(H1N1) exhibited very low acute-phase antibody levels.

**Fig. 5:**
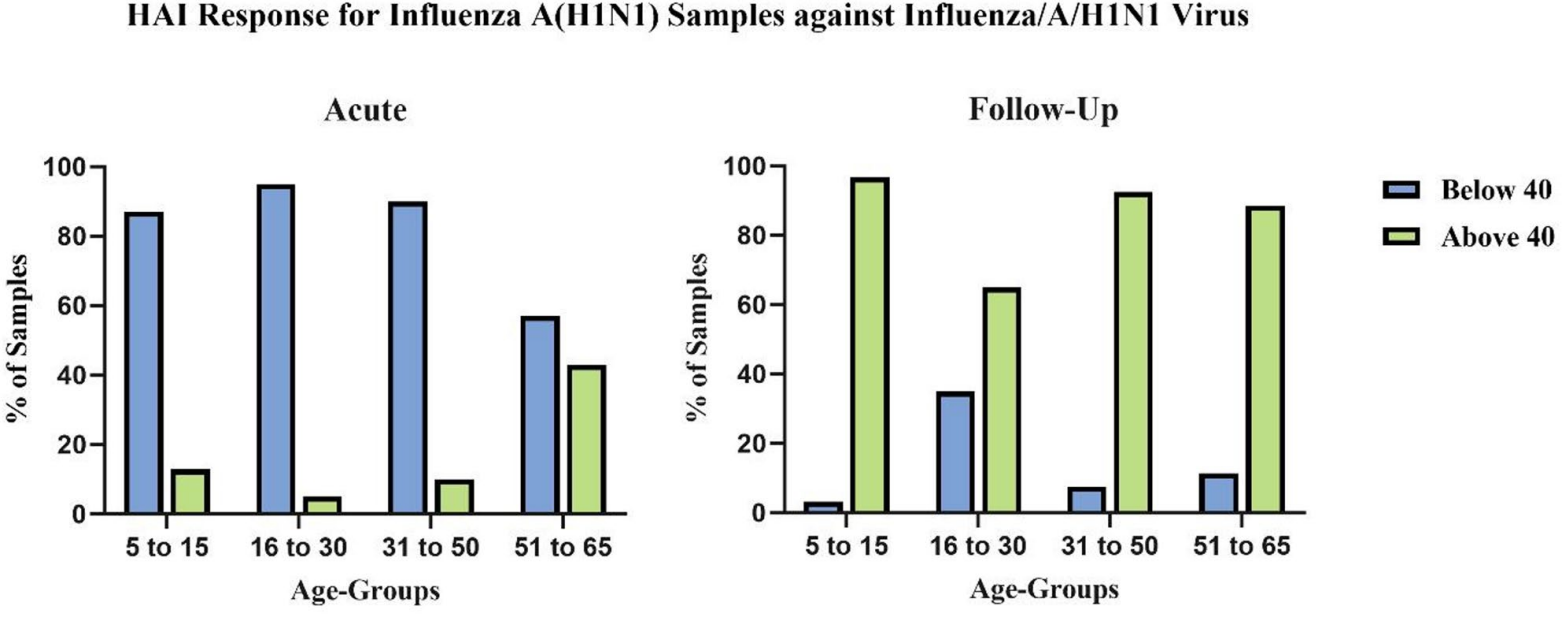
Age-stratified HAI Titer among Influenza A(H1N1)-positive samples: (**A**) acute samples and (**B**) follow-up samples. The X-axis shows age groups; the Y-axis shows the percentage of samples with HAI titers More than or equal to 40 (≥ 40) and below 40 (< 40)

Age-stratified analysis revealed considerable variation in baseline immunity and post infection responses (Fig. 5); in the acute phase, only 12% (2/31) of the 5–15 age group, 5% (2/40) of the 16–30 age group, 10% (4/40) of the 31–50 age group, and 42% (15/35) of the 51–65 age group had protective titers (HAI ≥ 40). In contrast, during the follow-up phase, 96.8% (30/31) of the 5–15-year age groups, 65% (26/40) of the 16–30-year age groups, 92.5% (37/40) of the 31–50-year age groups, and 88.6% (31/35) of the 51–65-year age groups presented HAI titers of ≥ 40. Additionally, a fourfold increase in antibody titer was observed in 122 individuals (83.5%), indicating a substantial increase in humoral immunity.

**Fig. 6:**
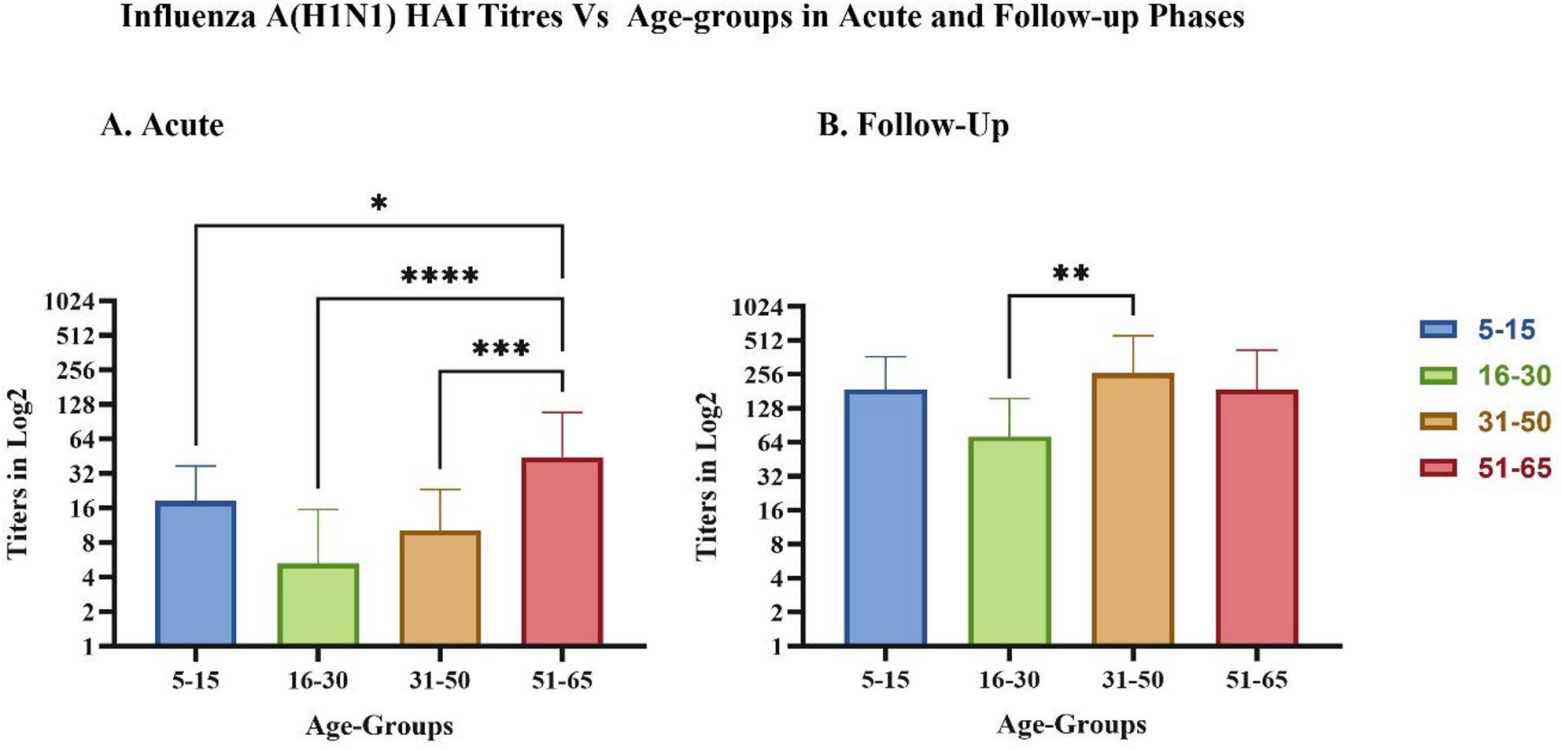
Hemagglutination inhibition (HAI) titers across age groups in influenza A(H1N1)-positive samples. Bar graphs display HAI titers (log₂ scale) stratified by different age groups in (**A**) acute-phase and (**B**) follow-up samples. Statistical significance between age groups was assessed using two-way ANOVA with Tukey’s multiple comparisons denoted by asterisks (*p < 0.05, **p < 0.01, ***p < 0.001, ****p < 0.0001)

Further age-stratified analysis of HAI titers is shown in Fig. 6. In the acute phase (Fig. 6A), titers were low in all the age groups, but in the follow-up samples (Fig. 6B), an increase in HAI titers was observed across all age groups, with several samples having titers as high as 1:1280, indicating robust antibody responses following infection. Two-way ANOVA with Tukey’s multiple comparisons revealed significant differences between age groups and sampling time points.

### Preexisting antibodies to influenza A(H3N2) and influenza A(H1N1) in acute samples

To assess preexisting immunity, acute-phase serum samples from patients positive for influenza A(H3N2) and A(H1N1) were analyzed on the basis of the time between symptom onset and sample collection (1–3 days versus ˃3 days). Individuals with HAI titers ≥ 40 within 1–3 days after symptom onset were considered to have preexisting antibodies, as these titers reflect prior exposure rather than an early immune response to the current infection.

**Fig. 7:**
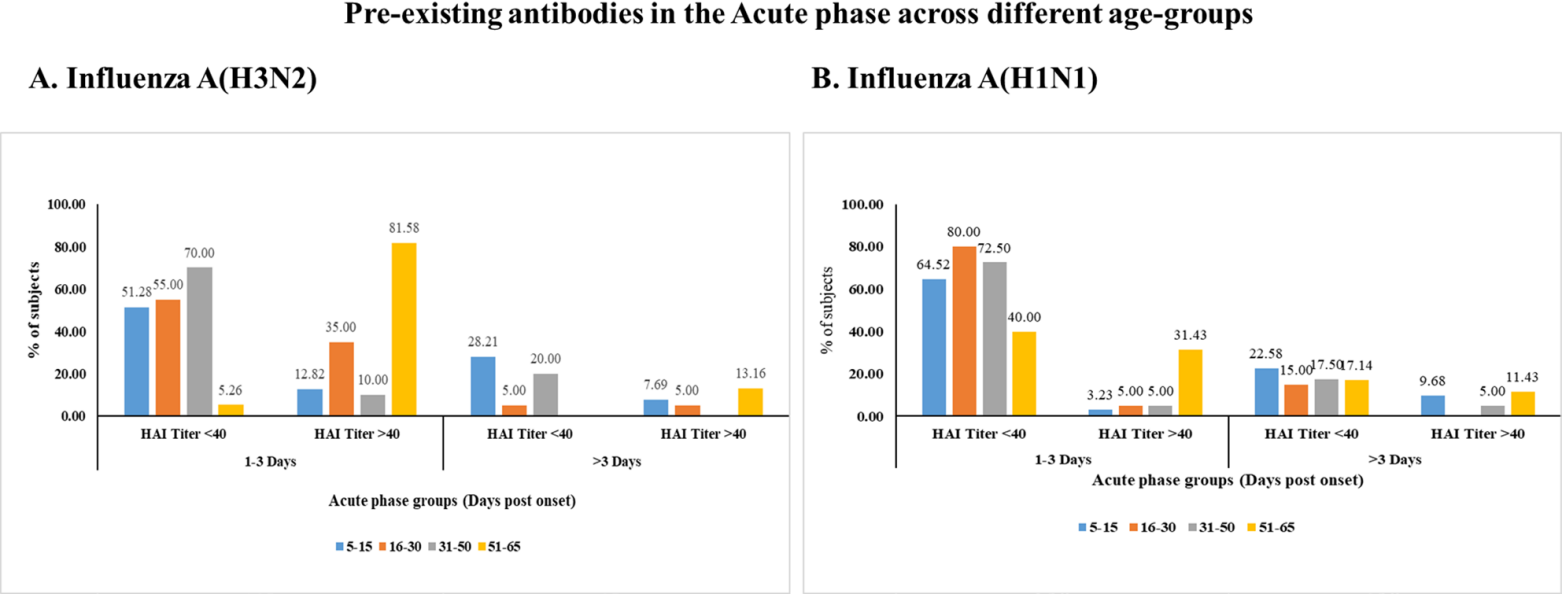
Comparison of HAI titers in influenza A(H3N2)- and influenza A(H1N1)-positive samples on the basis of days between symptom onset and sample collection. (**A**) Influenza A(H3N2) and (**B**) influenza A(H1N1) samples were stratified into two groups: 1–3 days and more than 3 days after symptom onset. The Y-axis represents the percentage (%) of samples, whereas the X-axis categorizes samples on the basis of HAI titers (below 40 and above 40) and age groups (5–15 years, 16–30 years, 31–50 years, and 51–65 years)

Among influenza A(H3N2)-positive patients, the highest proportion of preexisting antibodies was found in the 51–65-year-old age group, where 81.58% (31/38) had HAI titers ≥ 40 within 1–3 days after symptom onset. In contrast, only 12.82% (5/39) of the 5–15 age groups, 35% (14/40) of the 16–30 age groups, and 10% (4/40) of the 31–50 age groups presented titers ≥ 40, suggesting lower preexisting immunity in younger individuals (Fig. 7A).

With respect to influenza A(H1N1)-positive patients, preexisting immunity was lower across all age groups. The 51–65 age group had the highest seropositivity (31.43%, 11/35), followed by minimal rates in the 5–15 (3.23%, 1/31), 16–30 (5%, 2/40), and 31–50 (5%, 2/40) age groups, indicating limited prior exposure among younger participants (Fig. 7B).

### Sero-protection and seroconversion to influenza A(H3N2) and influenza A(H1N1)

**Fig. 8:**
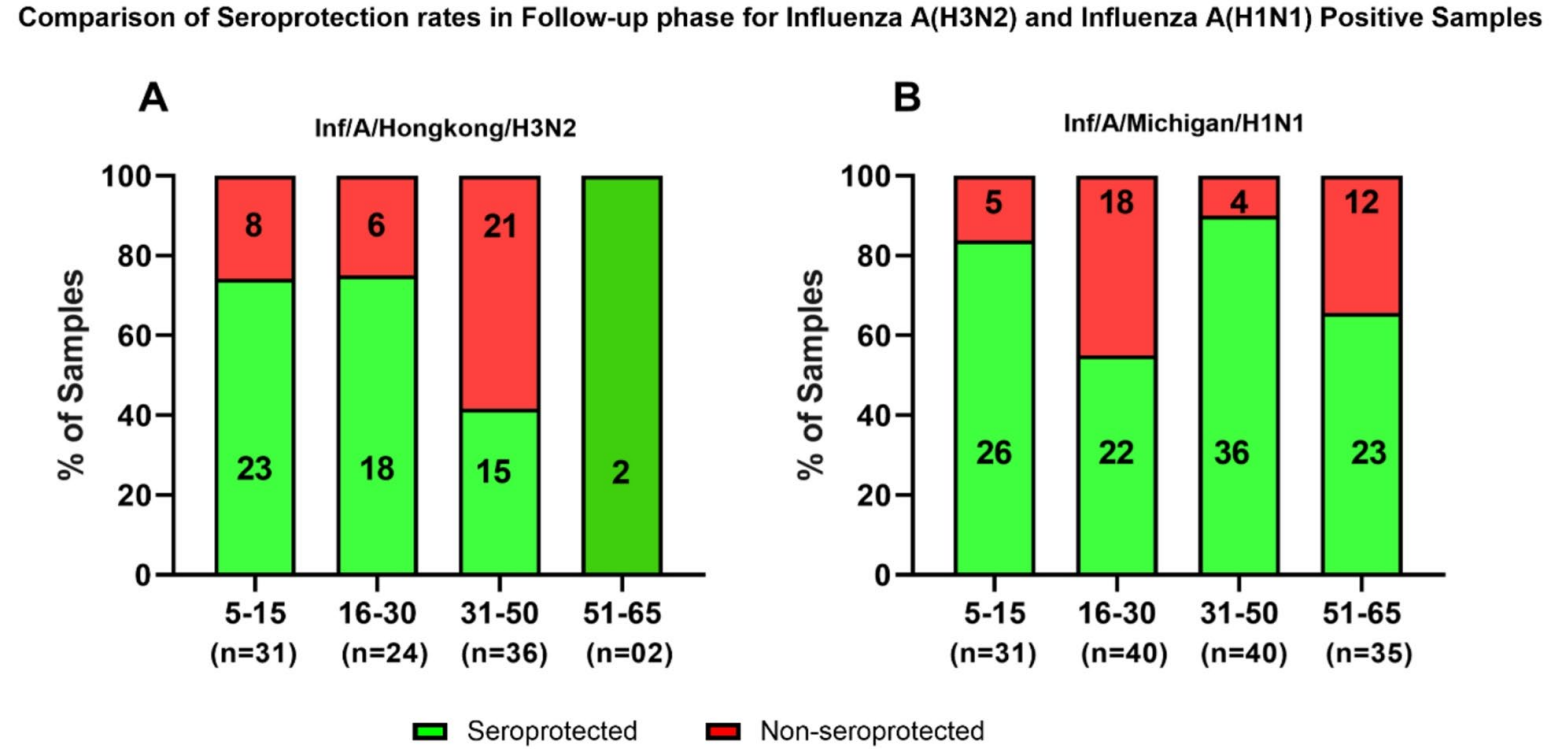
Age-stratified seroprotection following influenza infection in **A**) influenza A(H3N2) and **B**) influenza A(H1N1) infection. The figure displays the percentage of individuals with follow-up HAI titers ≥ 40 across four age groups (5–15, 16–30, 31–50, and 51–65 years). The green bars represent seroprotected individuals, and the red bars represent nonseroprotected individuals. The sample size (n) for each age group is shown below the X-axis

Sero-protection was defined as a follow-up HAI titer ≥ 40, and individuals not meeting this criterion were classified as nonseroprotected. Seroconversion was defined as a ≥ 4-fold increase in the HAI antibody titer between paired acute and follow-up samples. Age-stratified seroprotection proportions are presented in Fig. 8. Additionally, the geometric mean titers (GMTs) at both the acute and follow-up phases, fold-up values and other immune categories are summarized in Tables 2 and 3.

**Table 2:** Geometric mean titer (GMT) at acute and follow-up | Fold Rise | Seroconversion and Sero protection rates for influenza A(H3N2)-positive samples

Influenza A(H3N2) positive samples							
Age	*n*-value	Acute (GMT)	Follow-Up (GMT)	Fold-rise	Seroprotected With Seroconvertion *n* (%)	Seroprotected without Seroconversion *n* (%)	Neither *n* (%)
**5_to_15**	39	14.86	67.83	4.56	22 (56.4%)	9 (23.1%)	8 (20.5%)
**16_to_30**	40	31.09	90.75	2.91	21 (52.5%)	13 (32.5%)	6 (15%)
**31_to_50**	40	10.65	18.2	1.7	11 (27.5%)	7 (17.5%)	22 (55%)
**50_to_65**	38	56.57	117.13	2.07	12 (31.6%)	26 (68.4%)	0

**Table 3:** Geometric mean titer (GMT) at acute and follow-up | Fold Rise | Seroconversion and Sero protection rates for influenza A(H1N1)-positive samples

Influenza A(H1N1) positive samples							
Age	*n*-value	Acute (GMT)	Follow-Up (GMT)	Fold Rise	Seroprotected With Seroconvertion *n* (%)	Seroprotected without Seroconversion *n* (%)	Neither *n* (%)
5_to_15	31	14.3	125.11	8.74	26 (83.9%)	4 (12.9%)	1 (3.2%)
16_to_30	40	6.95	42.13	6.06	22 (55.0%)	4 (10%)	14 (35%)
31_to_50	40	9.49	151.89	16	36 (90.0%)	1 (2.5%)	3 (7.5%)
50_to_65	35	24.38	112.02	4.59	23 (65.7%)	8 (22.9%)	4 (11.4)

With respect to influenza A(H3N2), as shown in Fig. 8A, seroprotection was greatest among younger individuals in the 5–15-year-old age group, with 74.2% of the samples (23/31) being seroprotected. A similar trend was observed in the 16–30 age group, where 75% of the samples (18/24) were seroprotected. The seroprotection rate decreased significantly in the 31–50 year age group, where only 41.7% of the samples (15/36) were seroprotected. Among those aged 51–65 years, only 2/2 (100%) were seroprotected, as most individuals in this group already had HAI titers of ≥ 40 in the acute phase. *(*Fig. 8A*)*

Following natural infection with influenza A(H3N2), substantial age-related differences in immune response patterns were observed. Table 2 summarizes the geometric mean titers (GMTs), fold increases, seroconversion rates and seroprotection rates for influenza A(H3N2)-positive individuals. The 5–15-year-old group had the greatest increase (4.56-fold), with 56.4% (22/39) achieving both seroconversion and seroprotection, 23.1% (9/39) remaining seroprotected without seroconversion and 20.5% (8/39) being classified as neither. In the 16–30-year-old group (2.91-fold increase), 52.5% (21/40) were seroconverted and seroprotected, 32.5% (13/40) were seroprotected without seroconversion, and 15% (6/40) were neither. The 31–50-year-old group demonstrated the lowest increase (1.7-fold), with only 27.5% (11/40) achieving seroconversion and seroprotection, while 55% (22/40) were classified as neither. Among individuals aged 51–65 years (2.07-fold increase), 31.6% (12/38) were seroconverted and seroprotected, and 68.4% (26/38) were seroprotected without seroconversion; none were classified as neither. Notably, no individuals in this age group were classified as seroconverted or seroprotected, indicating protective antibody levels following infection.

In contrast, for the influenza A(H1N1) samples, a different pattern was observed. The highest seroprotection rates were recorded in the 31–50-year-old age group, with 90% (36/40) of individuals showing HAI titers ≥ 40 in the follow-up phase. In the 5–15-year-old age group, 83.9% (26/31) of the samples were seroprotected, indicating a strong immune response in younger individuals. In the 16–30-year-old age group, the degree of seroprotection was 55% (22/40), indicating a moderate response, whereas in the 51–65-year-old age group, 65.7% (23/35) of the samples demonstrated seroprotection (Fig. 8B).

With respect to influenza A(H1N1) pdm09 infection, seroconversion rates were higher across most age groups than among samples positive for influenza A(H3N2). Table 3 presents the corresponding data for individuals with influenza A(H1N1). The 5–15 year age group showed an 8.74-fold increase, with 83.9% (26/31) achieving seroconversion and seroprotection. In the 16–30-year-old group (6.06-fold), 55.0% (22/40) were seroconverted and seroprotected, whereas 35.0% (14/40) were neither. The 31–50-year-old group demonstrated the greatest increase (16-fold), corresponding to 90.0% (36/40) seroconversion and seroprotection. Among 51–65-year-olds (4.59-fold increase), 65.7% (23/35) achieved seroconversion and seroprotection, and 22.9% (8/35) were seroprotected without seroconversion.

### Preexisting antibodies against influenza A and influenza B strains

To further understand the presence of antibodies against heterologous influenza strains, individuals who were positive for influenza A(H3N2) and influenza A(H1N1) were evaluated for antibody responses against other circulating influenza subtypes and lineages. The study population was categorized on the basis of the presence of preexisting antibodies (acute phase titer ≥ 40) or the absence of preexisting antibodies (acute phase titer < 40) and their seroprotection status at the follow-up phase across different age groups. Descriptions of these responses are presented in Tables 4 and 5.

**Table 4:** Antibody responses against heterologous influenza strains among individuals seropositive for influenza A(H3N2)

Influenza A(H3N2) vs. Influenza A(H1N1)							
		Preexisting			Non-Preexisting		
**Age Group**	**Total n**	**Acute ≥ 40**	**Stayed Seroprotected in Follow-up (≥ 40)**	**GM Fold Rise**	**Acute < 40**	**Seroprotected in Follow-up (≥ 40)**	**GM Fold Rise**
5–15	39	30	26/30	0.74	9	5/9	29.3
16–30	40	7	5/7	1.6	33	7/33	1.4
31–50	40	1	0	0.5	39	7/39	1.2
51–65	38	3	2/3	1.1	35	6/35	1.4

**Influenza A(H3N2) vs. Influenza B Victoria**							
		**Preexisting**			**Non-Preexisting**		
**Age Group**	**Total n**	**Acute ≥ 40**	**Stayed Seroprotected in Follow-up (≥ 40)**	**GM Fold Rise**	**Acute < 40**	**Seroprotected in Follow-up (≥ 40)**	**GM Fold Rise**
5–15	39	9	7/9	0.7	30	2/30	1.4
16–30	40	11	6/11	0.6	29	3/29	1.1
31–50	40	2	1/2	0.7	38	0/38	1
51–65	38	5	2/5	0.5	33	4/33	1

**Influenza A(H3N2) vs. Influenza B Yamagata**							
		**Preexisting**			**Non-Preexisting**		
**Age Group**	**Total n**	**Acute ≥ 40**	**Stayed Seroprotected in Follow-up (≥ 40)**	**GM Fold Rise**	**Acute < 40**	**Seroprotected in Follow-up (≥ 40)**	**GM Fold Rise**
5–15	39	8	6/8	0.9	31	1/31	1
16–30	40	1	1/1	2	39	2/39	1.1
31–50	40	0	0/40	1	40	0/40	1
51–65	38	4	3/4	1.1	34	6/34	1.2

Distribution of antibody titers against influenza A(H1N1), influenza B Victoria, and influenza B Yamagata among individuals with influenza A (H3N2) antibodies at baseline, stratified by age group. Participants were categorized on the basis of the presence of preexisting antibodies (acute phase titer ≥ 40) and nonpre-existing antibodies (acute phase titer < 40). The table shows the number of individuals who remained seroprotected or achieved seroprotective titers (≥ 40) at follow-up. The GM fold increase represents the geometric mean fold increase in antibody titers between baseline and follow-up

**Table 5:** Antibody responses against heterologous influenza strains among individuals seropositive for influenza A(H3N2)

Influenza A(H1N1) vs. Influenza A(H3N2)							
		Preexisting			Non-Preexisting		
**Age Group**	**Total n**	**Acute ≥ 40**	**Stayed Seroprotected in Follow-up (≥ 40)**	**GM Fold Rise**	**Acute < 40**	**Seroprotected in Follow-up (≥ 40)**	**GM Fold Rise**
5–15	30	9	8/9	1	21	5/21	1.4
16–30	39	38	35/38	1.3	1	0/1	1
31–50	35	24	21/24	1.2	11	4/11	1.8
51–65	38	27	26/27	1.6	7	6/7	3.3

**Influenza A(H1N1) vs. Influenza B Victoria**							
		Preexisting			Non-Preexisting		
**Age Group**	**Total n**	**Acute ≥ 40**	**Stayed Seroprotected in Follow-up (≥ 40)**	**GM Fold Rise**	**Acute < 40**	**Seroprotected in Follow-up (≥ 40)**	**GM Fold Rise**
5–15	30	1	1/1	1	29	3/29	1.3
16–30	39	8	8/8	1	35	1/35	1.1
31–50	35	5	5/5	1.1	30	9/30	1.6
51–65	38	12	12/12	1.4	22	11/22	2.4

**Influenza A(H1N1) vs. Influenza B Yamagata**							
		Preexisting			Non-Preexisting		
**Age Group**	**Total n**	**Acute ≥ 40**	**Stayed Seroprotected in Follow-up (≥ 40)**	**GM Fold Rise**	**Acute < 40**	**Seroprotected in Follow-up (≥ 40)**	**GM Fold Rise**
5–15	30	3	3/3	1	27	3/27	1.1
16–30	39	4	4/4	1.2	35	6/35	1.4
31–50	35	5	5/5	1	30	3/30	1.4
51–65	38	13	12/13	1.5	21	9/21	1.9

Distribution of antibody titers against influenza A(H1N1), influenza B Victoria, and influenza B Yamagata among individuals with influenza A(H3N2) antibodies at baseline, stratified by age group. Participants were categorized on the basis of the presence of preexisting antibodies (acute phase titer ≥ 40) and nonpre-existing antibodies (acute phase titer < 40). The table shows the number of individuals who remained seroprotected or achieved seroprotective titers (≥ 40) at follow-up. The GM fold increase represents the geometric mean fold increase in antibody titers between baseline and follow-up

#### Antibodies against heterologous strains among influenza A(H3N2)-positive samples

Among individuals positive for influenza A(H3N2), acute and follow-up samples were measured against influenza A(H1N1), influenza B Victoria, and influenza B Yamagata viruses. (Table 4). With respect to antibodies against influenza A(H1N1), most individuals with acute-phase HAI titers ≥ 40 remained seroprotected at follow-up. In the 5–15-year age group, 26/30 individuals retained HAI titers ≥ 40 (GM fold increase: 0.74). Similarly, 5/7 individuals in the 16–30-year age group remained seroprotected (GM fold increase: 1.6). In the 31–50-year-old group, only a single individual with a baseline titer ≥ 40 did not retain seroprotective titers at follow-up (GM fold increase: 0.5), whereas 2/3 of the individuals in the 51–65-year-old group remained seroprotected (GM fold increase: 1.1).

Among individuals with acute-phase HAI titers < 40, very few samples developed seroprotective titers during follow-up. In the 5–15-year age group, 5/9 individuals achieved titers ≥ 40 (GM fold increase: 29.3). In the 16–30-year-old group, 7/33 individuals became seroprotected (GM fold increase: 1.4), whereas 7/39 individuals in the 31–50-year-old group (GM fold increase: 1.2) and 6/35 individuals in the 51–65-year-old group (GM fold increase: 1.4) reached seroprotective titers at follow-up.

A similar pattern was observed for antibodies against influenza B Victoria. As shown in Table 5, 7/9 individuals in the 5–15-year-old group, 6/11 individuals in the 16–30-year-old group, 1/2 individuals in the 31–50-year-old group, and 2/5 individuals in the 51–65-year-old group whose acute phase titers were ≥ 40 remained seroprotected at follow-up. Among individuals with acute phase titers < 40, 2/30, 3/29, and 4/33 individuals in the 5–15, 16–30, and 51–65 year groups, respectively, achieved seroprotective titers during follow-up.

For influenza B Yamagata, the persistence of antibodies was also observed among individuals with acute phase titers ≥ 40. Specifically, 6/8 individuals in the 5–15-year-old group, 1/1 individual in the 16–30-year-old group, and 3/4 individuals in the 51–65-year-old group retained seroprotective titers at follow-up. Among those with acute phase titers < 40, 1/31, 2/39, and 6/34 individuals in the 5–15, 16–30, and 51–65 year groups, respectively, developed titers ≥ 40 during follow-up.

#### Antibodies against heterologous strains among influenza A(H1N1)-positive samples

A similar analysis was conducted among individuals with influenza A(H1N1)-positive samples to evaluate antibody responses against influenza A(H3N2), influenza B Victoria and influenza B Yamagata (Table 5).

With respect to antibodies against influenza A(H3N2), most individuals with baseline titers ≥ 40 remained seroprotected at follow-up. In the 5–15-year-old age group, 8/9 individuals retained titers ≥ 40, whereas 35/38 individuals in the 16–30-year-old group, 21/24 individuals in the 31–50-year-old group, and 26/37 individuals in the 51–65-year-old group maintained seroprotective titers at follow-up. The overall fold increase ranged from 1 to 1.6 across age groups.

With respect to influenza B Victoria, individuals with acute-phase HAI titers ≥ 40 largely maintained seroprotective titers at follow-up across all age groups. As shown in Tables 5, 1/1 of individuals in the 5–15-year-old group, 8/8 individuals in the 16–30-year-old group, 5/5 individuals in the 31–50-year-old group, and 12/12 individuals in the 51–65-year-old group retained titers ≥ 40, with the increase in GM content ranging from 1.0 to 1.4.

Among individuals with acute-phase HAI titers < 40, a subset developed seroprotective titers during follow-up. Specifically, 3/29 individuals in the 5–15-year-old group, 1/35 individuals in the 16–30-year-old group, 9/30 individuals in the 31–50-year-old group, and 11/22 individuals in the 51–65-year-old group achieved HAI titers ≥ 40 at follow-up, with the increase in GM concentration ranging from 1.1 to 2.4.

For influenza B Yamagata, the persistence of antibodies was also observed among individuals with acute-phase HAI titers ≥ 40. As shown in Tables 5, 3/3 of the individuals in the 5–15-year-old group, 4/4 of the individuals in the 16–30-year-old group, 5/5 of the individuals in the 31–50-year-old group, and 12/13 of the individuals in the 51–65-year-old group remained seroprotected at follow-up. Among those with acute phase titers < 40, 3/27 individuals in the 5–15-year-old group, 6/35 individuals in the 16–30-year-old group, 3/30 individuals in the 31–50-year-old group, and 9/21 individuals in the 51–65-year-old group reached HAI titers ≥ 40 during follow-up.

## Discussion

The findings of this study offer insights into the clinical presentation and humoral immune response to influenza A(H3N2) and influenza A(H1N1) pdm09 among the Indian population. This study describes different patterns in symptom distribution, seroconversion rates, and preexisting immunity across age groups, offering a better understanding of host‒pathogen interactions and immune responses to seasonal influenza infections.

Both influenza A(H3N2) and influenza A(H1N1) pdm09 infections commonly present with cough, headache, and general weakness, although certain symptoms vary depending on the viral subtype. Coryza was more commonly reported among individuals positive for influenza A(H3N2), whereas individuals positive for influenza A(H1N1) pdm09 were more likely to experience myalgia and chills. Similar variations in symptom profiles between influenza subtypes have been reported in previous surveillance studies, where differences in viral pathogenicity and host immune responses influence the clinical manifestations of infection [21]. Age-related differences in symptoms were also highlighted; older adults (51–65 years) reported more severe symptoms, such as breathlessness and chest pain. These observations support the previous studies suggesting that age-associated immune changes and the presence of comorbidities may contribute to increased disease severity in the older population [22–24].

There is currently limited information on the antibody response following natural infection with influenza A(H3N2) and influenza A(H1N1) pdm09 in the Indian population. Our previous study by Alladi et al. found age-dependent differences in antibody responses to influenza A(H1N1) positive individuals [25]. However, this study was limited to a single state and involved a smaller sample size. In the present study, natural infection with influenza A(H3N2) and influenza A(H1N1)pdm09virus resulted in seroconversion within 4–6 weeks of illness, indicating the development of a humoral response. Seroconversion was observed in 77.1% of individuals infected with influenza A(H3N2) and 84.9% of individuals infected with influenza A(H1N1) pdm09. A similar study was reported by *Waalen* et al., in which 40% of the study population showed seroconversion [26].

In acute samples collected within three days of illness onset, 40.8% of individuals had HAI titers ≥ 40 for influenza A(H3N2), suggesting prior exposure to these viruses, whereas for influenza A(H1N1), only 17.1% of the population had HAI titers ≥ 40. The majority of individuals aged 51–65 years had preexisting antibodies, reflecting long-lasting immune memory [16]. Preexisting immunity reduces the severity of symptoms and limits viral spread, highlighting the importance of humoral responses in limiting the impact of new influenza strains. In line with observations by *Joshua* et al., this study examined how the timing of sample collection relative to illness onset affects antibody titers among influenza-positive patients. The early detection of antibodies soon after the onset of symptoms suggests that this reflects preexisting immune memory from previous influenza infection or vaccination, which has led to the maintenance of long-lasting B cells [27].

Age-stratified analysis of seroprotection and seroconversion revealed distinct immune response patterns following infection with influenza A(H3N2) and influenza A(H1N1)pdm09. Younger age groups showed strong immune responses to both influenza A(H3N2) and influenza A(H1N1). For influenza A(H3N2), the seroconversion rates were 56.4% in the 5–15 age group and 52.5% in the 16–30 age group. In contrast, for influenza A(H1N1), the seroconversion rates were 83.9% and 55.0% for the 5–15 and 16–30 age groups, respectively. These findings are consistent with those of previous studies suggesting that children and young adults have more adaptable immune systems and limited prior exposure to antigenically mismatched influenza strains, allowing them to mount strong responses [12, 13].

A marked difference in immunity was observed in the 31–50-year age group. In this age group, seroconversion rates were relatively low (27.5%) for influenza A(H3N2) because of waning humoral immunity or the production of non-neutralizing antibodies. In contrast, 90% of the samples in the same age group demonstrated seroconversion for influenza A(H1N1), with a 28-fold increase in the HAI titer, reflecting a better immunological response against this subtype. According to previous studies, the middle-aged population is continuously at risk of contracting the influenza A(H3N2) virus, which in turn elicits nonneutralizing antibodies. They may exhibit a modest HAI response but a significant nonneutralizing immune response, such as cellular immune responses and antibodies against the HA stalk and neuraminidase (NA). This nonneutralizing immunity, which was not evaluated in our study, may prevent infections and produce strain-specific responses [28].

Older adults presented high baseline antibody levels and significant seroconversion for both subtypes. For influenza A(H3N2), 100% of the follow-up samples had HAI titers ≥ 40, suggesting strong cumulative immunity from repeated exposure to antigenically similar strains. However, the increase in the HAI titer was modest compared with that in younger age groups. For influenza A(H1N1), seroconversion was also high at 73.53%, supported by substantial baseline titers, indicating widespread prior exposure to this pandemic strain. Similar findings were reported by Waalen et al., who reported that older adults retain strong humoral immunity because of long-term antigenic exposure [15, 19].

Analysis of antibody responses against other influenza subtypes and lineages among individuals positive for influenza A(H3N2) and influenza A(H1N1) revealed the presence of detectable antibodies to heterologous influenza strains across different age groups. A substantial proportion of individuals with acute-phase HAI titers ≥ 40 retained seroprotective titers at follow-up, suggesting the persistence of antibodies that may reflect prior exposure to related influenza viruses circulating in the population. This persistence of seroprotective titers was observed across multiple age groups for both influenza A and influenza B lineages.

In addition, a subset of individuals who did not have preexisting antibodies at the acute phase (HAI titer < 40) developed seroprotective titers (HAI ≥ 40) at follow-up against these heterologous strains. While the mechanisms underlying this increase cannot be definitively determined in the present study, these observations may indicate either an increase in low-level antibodies from prior exposure or the development of antibodies capable of recognizing heterologous influenza strains. Similar patterns have been reported in influenza seroepidemiological studies in which antibody responses against one influenza subtype are associated with measurable responses against other subtypes or lineages [29, 30].

Seasonal influenza vaccination is not a routine or widespread public health practice in the regions of India covered in this study. Therefore, the antibody response patterns observed in this study reflect the development of immunity following natural infection and previous exposure to circulating influenza viruses rather than vaccine-induced immunity. The viral strains used for serological analyses in this study corresponded to those circulating in India during the study period, including A(H1N1)pdm09 Michigan/2015-like and A(H3N2) Hong Kong/2014-like and Singapore/2016-like viruses, which is consistent with the WHO-recommended vaccine strains for the 2016–2017 Northern Hemisphere influenza season (WHO Influenza Vaccine Recommendations, 2016) [31].

This study has certain limitations: the number of samples available within each age group was relatively small, which may limit the ability to detect more subtle age-dependent differences in immune responses. Additionally, antibody responses in this study were assessed using the hemagglutination inhibition (HAI) assay; other important components of influenza immunity, including non-neutralising antibodies, antibodies targeting the HA stalk or neuraminidase proteins, and cell-mediated immune responses, were not evaluated in this study. Future investigations incorporating broader immunological assays and larger study populations will help provide a more comprehensive understanding of immune responses following influenza infection in the Indian population and aid in the development of strategies to prevent severe illness, especially in at-risk age groups.

## Conclusion

Overall, the results suggest that while both influenza A(H3N2) and influenza A(H1N1) infections can elicit strong immune responses, the degree and patterns of these responses vary significantly. The stronger response observed in younger age groups for influenza A(H1N1) could reflect their immune system’s capacity to respond more vigorously to this strain. In contrast, older influenza A(H3N2) patients exhibit a distinct immune response, potentially because of previous exposure. These findings contribute to understanding the variable immune responses elicited by different influenza subtypes and could inform targeted public health strategies and vaccine development.

## Supplementary Information

Supplementary Material 1

## Data Availability

All the data relevant to this study will be available request from the corresponding author upon request.
