# Supplementary material for: Assessment of pre and postinfection immunity to the hemagglutinin proteins of Influenza A(H1N1)pdm09 and Influenza A(H3N2) in India: a retrospective longitudinal study
Source: Virol J. 2026 Apr 5;23:141. doi: 10.1186/s12985-026-03146-w (PMC13220398; doi:10.1186/s12985-026-03146-w)
Supplement: Supplementary file 1 — Supplementary Material 1 [file 12985_2026_3146_MOESM1_ESM.docx]

SUPPLEMENTARY DATA

*Supplementary Table 1: Age-wise distribution of clinical symptoms in influenza A(H1N1) and influenza A(H3N2) positive samples. The table shows the prevalence (%) of each symptom across age groups, with p values from chi-square tests indicating the statistical significance of age-related differences within each virus type.*

| **Clinical parameters** | **Influenza A(H1N1) Positive Samples** | | | | | **Influenza A(H3N2) Positive Samples** | | | | | |
| --- | --- | --- | --- | --- | --- | --- | --- | --- | --- | --- | --- |
|  | **Age-Groups** | | | | | **Age-Groups** | | | | | |
|  | **5 to 15** | **16 to 30** | **31 to 50** | **51 to 65** | **p-values** | **5 to 15** | **16 to 30** | **31 to 50** | **51 to 65** | **p-values** |  |
|  | **n/N (%)** | **n/N (%)** | **n/N (%)** | **n/N (%)** |  | **n/N (%)** | **n/N (%)** | **n/N (%)** | **n/N (%)** |  |  |
| **Chills** | 29/32 (90.6) | 33/40 (82.5) | 29/40 (72.5) | 26/35 (74.3) | 0.217 | 24/39 (61.5) | 31/40 (77.5) | 32/40 (80) | 24/38 (63.2) | 0.159 |  |
| **Night sweats** | 16/32 (50) | 24/40 (60) | 26/40 (65) | 23/35 (65.7) | 0.528 | 20/39 (51.3) | 20/40 (50) | 28/40 (70) | 20/38 (52.6) | 0.231 |  |
| **Coryza** | 23/32 (71.9) | 30/40 (75) | 25/40 (62.5) | 21/35 (60) | 0.449 | 33/39 (84.6) | 32/40 (80) | 32/40 (80) | 32/38 (84.2) | 0.914 |  |
| **Cough** | 29/32 (90.6) | 37/40 (92.5) | 40/40 (100) | 31/35 (88.6) | 0.215 | 37/39 (94.9) | 34/40 (85) | 35/40 (87.5) | 34/38 (89.5) | 0.541 |  |
| **Sore throat** | 14/32 (43.8) | 17/40 (42.5) | 18/40 (45) | 21/35 (60) | 0.412 | 11/39 (28.2) | 13/40 (32.5) | 25/40 (62.5) | 16/38 (42.1) | 0.0095 |  |
| **Breathlessness** | 0/32 (0) | 5/40 (12.5) | 5/40 (12.5) | 7/35 (20) | 0.083 | 3/39 (7.7) | 5/40 (12.5) | 9/40 (22.5) | 6/38 (15.8) | 0.3 |  |
| **Chest pain** | 1/32 (3.1) | 6/40 (15) | 7/40 (17.5) | 6/35 (17.1) | 0.267 | 4/39 (10.3) | 3/40 (7.5) | 8/40(202) | 8/38 (21.1) | 0.221 |  |
| **Headache** | 28/32 (87.5) | 38/40 (95) | 35/40 (87.5) | 31/35 (88.6) | 0.647 | 34/39 (87.2) | 29/40 (72.5) | 35/40 (87.5) | 37/38 (97.4) | 0.0166 |  |
| **Myalgia** | 20/32 (62.5) | 35/40 (87.5) | 36/40 (90) | 30/35 (85.7) | 0.0104 | 21/39 (53.8) | 27/40 (67.5) | 36/40 (90) | 33/38 (86.8) | 0.00046 |  |
| **Joint Pain** | 9/32 (28.1) | 19/40 (47.5) | 25/40 (62.5) | 26/35 (74.3) | 0.001 | 13/39 (33.3) | 13/40 (32.5) | 26/40 (65) | 22/38 (57.9) | 0.004 |  |
| **General Weakness** | 26/32 (81.3) | 38/40 (95) | 35/40 (87.5) | 35/35 (100) | 0.0337 | 34/39 (87.2) | 35/40 (87.5) | 34/40 (85) | 36/38 (94.7) | 0.563 |  |
| **Nausea** | 16/32 (50) | 21/40 (52.5) | 16/40 (40) | 20/35 (57.1) | 0.492 | 20/39 (51.3) | 16/40 (40) | 19/40 (47.5) | 15/38 (39.5) | 0.662 |  |
| **Vomiting** | 14/32 (43.8) | 15/40 (37.5) | 7/40 (17.5) | 13/35 (37.1) | 0.085 | 13/39 (33.3) | 11/40 (27.5) | 14/40 (35) | 7/38 (18.4) | 0.365 |  |

**Wilcoxon test between acute and follow-up samples of influenza A(H3N2) and influenza A(H1N1) patients**


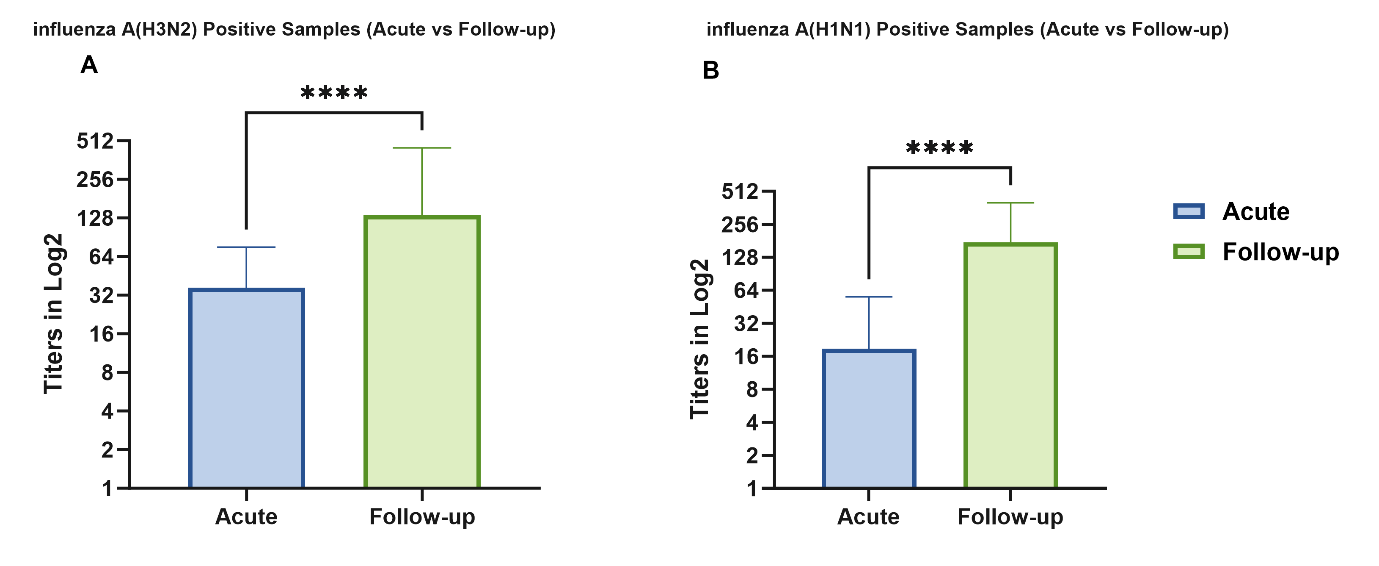


*Supplementary Figure 1: Hemagglutination inhibition (HAI) titres in influenza A positive samples at acute and follow-up time points.
Bar graphs showing HAI titres (log₂ scale) for individuals with (A) influenza A(H3N2) and (B) influenza A(H1N1) positive samples, comparing acute-phase and follow-up samples. The x-axis represents the sample collection time point (acute vs. follow-up), and the y-axis shows HAI titres in log₂ values. Statistical analysis was performed via the Wilcoxon matched-pairs signed-rank test. **** denotes p < 0.0001, indicating a highly significant increase in antibody titres for both influenza subtypes at the follow-up time point.*

**HAI response of influenza A(H3N2) virus:**


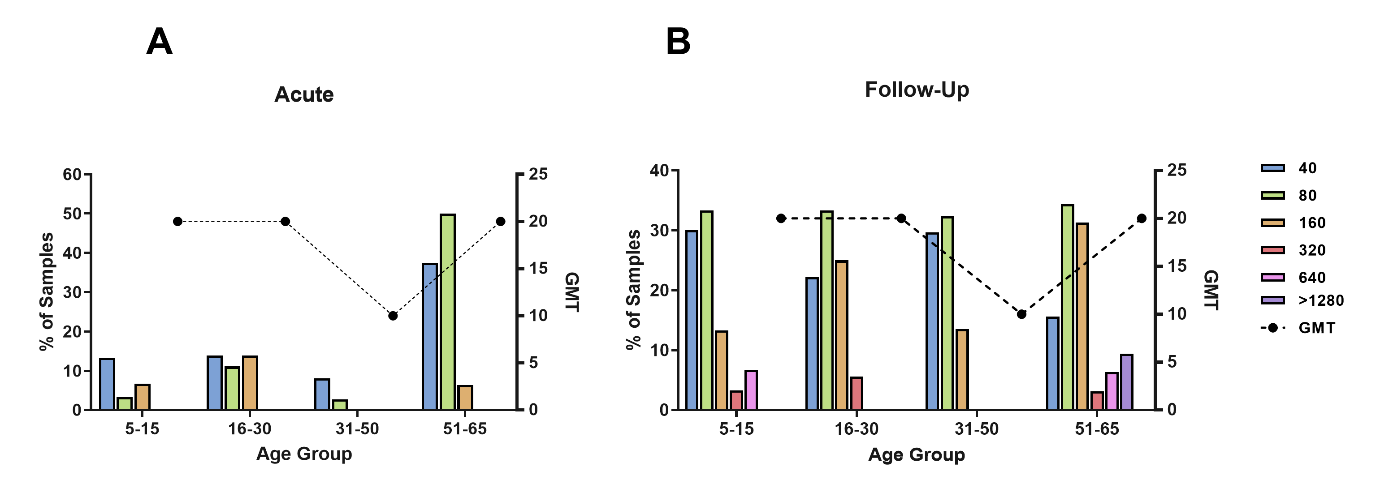


*Supplementary Figure 2: Age groupwise HAI titre against influenza A(H3N2) virus: (A) Acute samples. (B) Follow-up samples. X-axis represents age groups, Y-left axis represents the number of samples with HAI titres, and Y-right axis represents GMT.*

**HAI response of influenza A(H1N1) virus:**


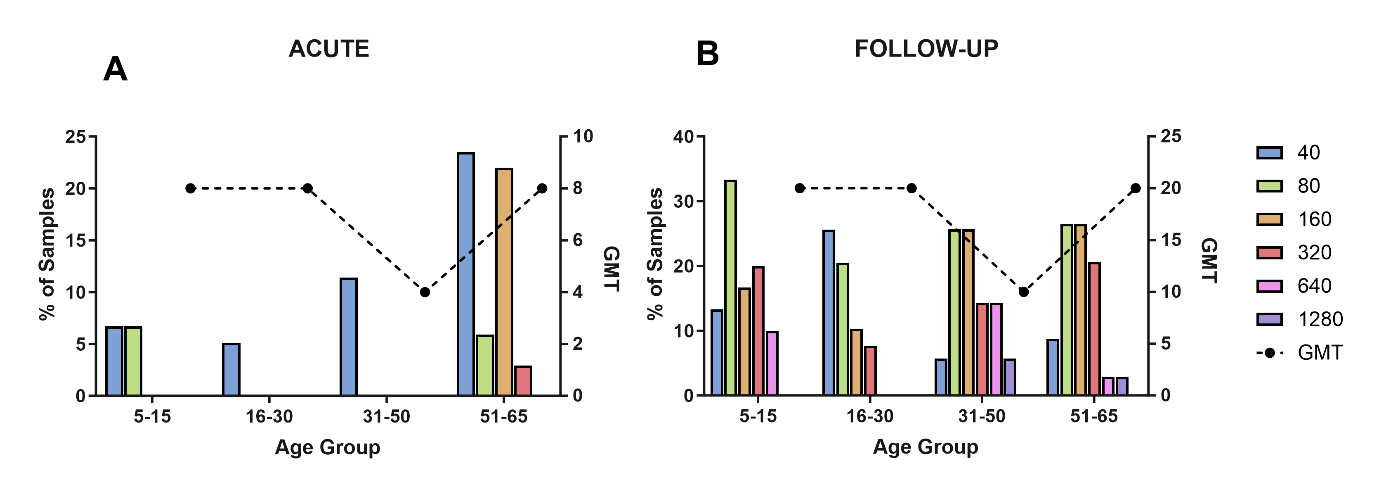


*Supplementary Figure 3: Age groupwise HAI titre against influenza A(H1N1) virus: (A) Acute samples. (B) Follow-up samples. X-axis represents age groups, Y-left axis represents the number of samples with HAI titres, and Y-right axis represents GMT.*
